# Determinants of early sexual initiation among female youth in Ethiopia: a multilevel analysis of 2016 Ethiopian Demographic and Health Survey

**DOI:** 10.1186/s12905-020-01069-4

**Published:** 2020-09-14

**Authors:** Mastewal Arefaynie, Melaku Yalew, Yitayish Damtie, Bereket Kefale

**Affiliations:** Department of Reproductive and Family Health, School of Public Health, College of Medicine and Health Sciences, Wollo University, PO Box: 1145, Dessie, Ethiopia

**Keywords:** Early sexual initiation, Female youth, Ethiopia, Multi-level analysis, EDHS 2016

## Abstract

**Background:**

Evidences on determinants of early sexual initiation among female youth is still limited especially; community-level factors are not investigated in Ethiopia. Therefore, the aim of this study was to assess individual and community-level factors associated with early sexual initiation among female youth in Ethiopia.

**Methods:**

The 2016 Ethiopian Demographic and Health Survey (EDHS) dataset were used and a total of 6143 participants (female youth) were included. Multi-level mixed-effect logistic regression was done to identify individual and community-level factors. Adjusted odds ratio along with 95% confidence interval was used to show the strength and direction of the association. Finally, the level of statistical significance was declared at *P* value less than 0.05.

**Results:**

Individual-level factors significantly associated with early sexual initiation among female youth were; age group from 19 to 24 years [AOR = 5.8, 95% CI = (4.6, 7.3)], not attending school [AOR = 14.1, 95% CI = (8.1, 24.7)], ever chewing Chat [AOR = 2.0, 95% CI = (1.3, 3.0)]. From community-level factors: living in Addis Ababa [AOR = 0.3, 95% CI = (0.2, 0.5)], living in Gambella [AOR = 2.7, 95% CI = (1.7, 4.3)] and live in a low proportion of poor communities [AOR = 0.7, 95% CI = (0.5, 0.9)] were significantly associated with early sexual initiation among female youth in Ethiopia.

**Conclusions:**

Age, low educational status, ever chewing Chat, region and live in a high proportion of poor community had a statistical association with early sexual initiation among female youth in Ethiopia. Improving educational coverage and community-level of wealth status are important intervention areas to delay the age of early sexual initiation.

## Background

Even though different countries and organizations have different age classification of youth, WHO, UNFPA and UNICEF classify individuals from age 10–19, 15–24 and 10–24 as adolescent, youth and young respectively. It is the transitional stage from childhood to adulthood with biological, social, psychological change [1, 2]. Many adult mental processes are started during this time. So, it is a time of risk and opportunity for their future life [3–6].

Different scholars define early sexual initiation according to the social and demographic context of the nation [7–9]. But, the Universal Declaration of Human Rights proclaimed as age below 18 years old is considered as a child, they couldn’t decide concerning marriage and/or consensual sexual relationship [10]. They are not ready in all mental, physical and social challenges and requirements for safe sexual practice and gestation. In Ethiopia, the minimum age of marriage is still 18 years [10] and sexual initiation before 18 years old is prohibited by law [11]. In addition, due to the cultural and religious tightness of the nation, Ethiopians initiated sexual activity after marriage. Despite the above assumption and legal issues, more than 60% of women start their sexual intercourse before they celebrate their 18 birth date [3, 5, 11–15].

Early sexual initiation has negative health, social and economic consequences for both the women and feature generation. It was a risk factor for sexually transmitted infections including HIV/AIDS [16–19], unsafe sexual practice [8, 9, 20], unwanted pregnancy [18, 21–23], mental problem and maternal death [16, 18, 24, 25]. It also increased the risk of school dropout, poor school performance, stigma and discrimination [26–28]. It also affects the social and economic status during adulthood [29].

In Ethiopia, different researches have been done on the prevalence and/or factors associated with early sexual initiation in female adolescent and youth. Age, residence, educational status, parent- youth discussion, using addictive substances and religion are determinant factors identified by scholars [3, 5, 11, 12, 14, 15, 30, 31]. But, all the studies were done at the local level, use a small sample size and do not consider the effect of community-level factors. Besides, the association at the individual-level may not work at the community-level and vice versa. Even all the studies were fitted with standard logistic regression which may lead them to loss of power. National representative evidence is important to achieve national and international goals. Therefore, this study aimed to assess individual and community-level factors associated with early sexual initiation among female youth in Ethiopia by using EDHS 2016 which will be important to develop community-level information education communication and behavioral change communication to reduce the prevalence and impact of early sexual initiation in the country.

## Methods

### Study setting and population

The study was conducted in Ethiopia, which is located in the North-eastern Africa which lies between 3^0^ and 15^0^ North latitude and 33^0^ 48^0^ and East longitudes. This study used the 2016 EDHS dataset which was conducted by the Central Statistical Agency (CSA) in collaboration with the Federal Ministry of Health (FMoH) and the Ethiopian Public Health Institute [32]. A cross-sectional study design using secondary data analysis of the 2016 EDHS was done among all-female youth (15–24 years old) irrespective of their sexual activity.

A total of 6143 females were included in the analysis. EDHS 2016 sample was stratified and selected in two stages. In the first stage, stratification was conducted by region and then each region stratified as urban and rural, yielding 21 sampling strata. A total of 645 (202 urban and 443 rural) enumeration areas (EAs) were selected proportionally to the size of EA in each sampling stratum. In the second stage, a fixed numbers of (28 households per cluster) were selected systematically from the households listed.

### Variable measurement

In this study, the outcome variable (early sexual initiation) was dichotomized as (Yes/No). Youth who started sexual activity at or before 18 years old considered as having early sexual initiation and those who started after 18 years old or not yet started during their youth time were considered as not having early sexual initiation [33]. The independent variables were either individual-level factors including (age, religion, chat chewing, drinking alcohol, wealth index, educational status, media exposure) or community-level factors (region, residence, community-level of education, community-level wealth index, community-level television exposure and community-level radio exposure) were created by aggregating individual-level factors in each cluster. The community-level of wealth index was generated by using the proportion of the two (poorest and poorer) the lowest level of wealth index to the total wealth index of the same cluster. Similarly, community level of education was generated by using the proportion of the two (no education and primary education) lowest level of educational attainment to the total educational level of the same cluster. The community level of television exposure was also computed by dividing not exposed at all to television for total television exposure. The community level of radio exposure was computed by dividing not exposed for radio at all to the total radio exposure. Since all the above four variables were not normally distributed, the median was used as a cutoff point.

### Data quality control, processing and analysis

Sample weighting was done to compensate the unequal probability of selection between the strata. The data were also collected after pre-test and necessary modifications were done. In addition, all the questionnaires were translated to local language of respondents. The SVY command was used to adjust for the complex sampling design. Data cleaning was conducted to check for the consistency with the EDHS-2016 descriptive report. Recoding, variable generation, labeling and analysis were done by using STATA/SE version 14.0. Descriptive statistics were done to describe the study participants in relation to socio-demographic characteristics. Multilevel analysis (a two-level mixed-effects logistic regression model) was conducted (ICC = 22.5%) since EDHS data are hierarchical (individual “level 1” variables were nested within community “level 2” variables). The log of the probability of early sexual initiation was modeled as follows [34]: Log [$\frac{\Pi \mathrm{ij}}{1-\Pi \mathrm{ij}}\kern0.5em \Big]$ = β_0_ + β_1_X_ij_ + B_2_ Z_ij_ + μ_j_ + e_ij_.

Where i and j are individual-level and community-level unites respectively; X and Z refer to individual and community-level variables respectively; *πij* is the probability of early sexual initiation for the i^th^ youth in the j^th^ community; β’s indicates the fixed coefficients. (Β_0_) is the intercept, μj showed the random effect and eij showed random errors. During analysis, both bi-variable and multivariable multilevel logistic regression was fitted. Variables with a *p* value less than 0.2 at model I and model II were selected to the final model. The analysis was done in four models. The first model (without explanatory variable), the second model (only individual-level variable), the 3rd (only community level variable) and the last model (both community level and individual level variables).

The likelihood of early sexual initiation among female youth and different explanatory factors were measured by Adjusted Odds Ratio (AOR) with respective 95% confidence level. Variables with p- value less than 0.05 at model-III were considered as significantly associated. The random-effects (variations) were measured by using ICC, Median Odds Ratio (MOR) and Proportional Change in Variance (PCV). ICC shows the variation in early sexual initiation among female youth due to community characteristics. It can be calculated as: ICC= $\left(\frac{\delta^2}{\delta^2+\frac{\pi^2}{3}}\right)$, where δ^2^ indicates the estimated variance of clusters. MOR is the median value of the odds ratio between the area at highest risk and the area the lowest risk when randomly picking out two areas and it was calculated as: MOR = exp. ($\sqrt{2\times {\delta}^2+.6745}$ ) ≈ exp.^(0.95δ)^. PCV measures the total variation attributed to individual-level variables and community-level variables in the final model (model-III) [35]. Multicollinearity among explanatory variables was checked by using standard error at cutoff point ±2 and there is no multicollinearity. The log-likelihood test was used to estimate the goodness of fit of the adjusted final model in comparison to the preceding models.

## Results

### Characteristics of the respondents

A total of 6143 female youth were included in the analysis. Among this, 3383 (52.8%) were found in the age group of 19–24 years, 1889 (29.5%) study participants were completed secondary and higher education. Three thousand eight hundred forty-five (60.1%) of female youth had no exposure to television. About 4676 (76.1%) of youth have resided in rural area (Table 1).

**Table 1:** Individual and community-level characteristics of female youth in Ethiopia, EDHS 2016 (*n* = 6143)

Variable	Number	Percent
**Age in years**		
15–18	3018	47.2
19–24	3383	52.8
**Religion**		
Orthodox	2613	40.8
Muslim	2569	40.1
Others^a^	1219	19.1
**Educational status**		
No education	1408	22.0
Primary	3104	48.5
Secondary	1361	21.3
Higher	528	8.2
**House hold Wealth index**		
Poorest	1571	24.6
Poorer	1051	16.4
Middle	1183	18.5
Richer	1141	17.8
Richest	1455	22.7
**Frequency of watching television**		
Not at all	3845	60.1
Less than once a week	805	12.6
At least once a week	1751	27.3
**Frequency of listening to radio**		
Not at all	4017	62.8
Less than once a week	1176	18.3
At least once a week	1208	18.9
**Ever heard about STI**		
No	457	7.1
Yes	5944	92.9
**Ever chewing chat**		
No	6024	94.1
Yes	377	5.9
**Ever drinking alcohol**		
No	4496	70.2
Yes	1905	29.8
**Residence**		
Urban	1467	23.9
Rural	4676	76.1
**Region**		
Tigray	498	8.1
Afar	56	0.9
Amhara	1382	22.5
Oromia	2229	36.3
Somali	186	3.0
Benishangul	67	1.1
SNNP	1251	20.4
Gambela	18	0.3
Harari	16	0.3
Addis Ababa	403	6.5
Dire Dawa	37	0.6
**Community level of wealth**		
Low	3159	51.4
High	2984	48.6
**Community level of education**		
Low	2827	46.0
High	3316	54.0
**Community level of television exposure**		
Low	2801	45.6
High	3342	54.4
**Community level of radio exposure**		
No	3350	54.5
Yes	2793	45.5

^a^protestant, catholic, traditional followers

### Individual and community-level factors associated with early sexual initiation

In the final model age, educational status, ever chewing Chat, region and community-level wealth had a statistical association with early sexual initiation. The odds of early sexual initiation was 6 times more among participants aged between 19 and 24 years as compared to their counterparts [AOR = 5.8, 95% CI = (4.6, 7.3)]. Female youth who were no attending school were 14 times more likely initiate sex at or before age 18 than attending higher education [AOR = 14.1, 95% CI = (8.1, 24.7)]. Female youth who ever chew Chat were 2 times more likely to initiate sex early as compared to not [AOR = 2.0, 95%CI = (1.3, 3.0)]. Female youth who were living in Addis Ababa were 70% less likely to initiate sex early as compared to youth living in Tigray region [AOR = 0.3, 95%CI = (0.2, 0.5)]. Likewise, female youth who were living in Gambella region were 3 times more likely to initiate sex early as compared to youth who living in Tigray region [AOR = 2.7,95%CI = (1.7, 4.3)]. Female youth who live in low proportion of poor communities were 30% less likely to initiate sex early as compared to female youth who live in a high proportion of poor community [AOR = 0.7, 95% CI = (0.5, 0.9)] (Table 2).

**Table 2:** multilevel logistic regression model for factors associated with early sexual initiation among female youth in Ethiopia, EDHS 2016 (*n* = 6143)

Variable	COR (95% CI)	Model-0 ICC = 22.6%	Model-I AOR (95% CI)	Model-II AOR (95% CI)	Model-III (AOR) (95% CI)
**Age in years**					
15–18	1				
19–24	5.1(4.1, 6.4)		5.6(4.5, 7.0)		**5.8 (4.6, 7.3)**
**Religion**					
Orthodox	1				
Muslim	1.6 (1.2, 2.0)		1.3 (0.9, 1.8)		1.35 (0.9, 1.9)
Others	0.8(0.5, 1.1)		0.8 (0.6, 1.1)		1.1(0.8, 1.5)
**Educational status**					
No education	9.4(5.8, 15.3)		14.7 (8.5, 25.4)		**14.1 (8.1, 24.7)**
Primary	2.7(1.7, 4.3)		5.9(3.5, 9.9)		**5.9 (3.5, 10.0)**
Secondary	1.3 (0.8, 2.2)		2.3 (1.4, 3.9)		**2.3 (1.4, 3.9)**
Higher	1				
**Household wealth index**					
Poorest	2.4 (1.6, 3.4)		1.1 (0.6, 1.8)		0.8 (0.5, 1.5)
Poorer	2.5 (1.8, 3.5)		1.1 (0.7, 1.8)		0.9 (0.6, 1.5)
Middle	2.5 (1.9, 3.5)		1.2 (0.7, 1.9)		1.1 (0.7, 1.7)
Richer	1.3(0.9, 1.8)		0.8 (0.5, 1.2)		0.71 (.46, 1.1)
Richest	1				
**Watching television**					
Not at all	1.8 (1.3, 2.4)		0.9 (0.6, 1.4)		0.9 (0.6, 1.4)
Less than once a week	1.3 (0.9, 1.8)		1.1 (0.7, 1.5)		0.95 (0.7, 1.4)
At least once a week	1				
**Listening radio**					
Not at all	1.1 (0.9, 1.5)		0.9 (0.6, 1.2)		0.9 (0.6, 1.2)
Less than once a week	0.8 (0.6, 1.0)				0.8 (0.5, 1.1)
At least once a week	1				
**Ever heard about STI**					
No	1				
Yes	1.1 (0.7, 1.6)		1.4 (0.9, 2.3)		1.4 (0.9, 2.2)
**Ever chewing chat**					
No	1				
Yes	2.5 (1.7, 3.6)		2.0 (1.4, 3.0)		**2.0 (1.3, 3.0)**
**Ever drinking alcoho**l					
No	1				
Yes	1.2 (1.0, 1.5)		1.4 (1.1, 1.8)		1.3 (0.9, 1.7)
**Residence**					
Urban	1				
Rural	2.8 (2.19, 3.47)			0.9 (0.6, 1.4)	1.1(0.7, 1.9)
**Region**					
Tigray	1				
Afar	2.4(1.6, 3.7)			1.0 (0.7, 1.5)	0.9 (.5, 1.5)
Amhara	1.2 (0.8, 1.8)			1.1 (0.8, 1.6)	1.1(0.7, 1.6)
Oromia	0.9 (0.6, 1.4)			0.7 (0.5, 1.1)	**0.6 (0.4, 0.9)**
Somali	1.2 (0.8, 1.8)			0.5 (0.3, 0.8)	**0.5 (0.3,0.8)**
Benishangul	1.2 (0.8, 1.8)			1.1 (0.7, 1.7)	1.1 (0.7, 1.8)
SNNP	0.5(0.4, 0.8)			0.4 (0.3, 0.6)	**0.4 (0.3, 0.7)**
Gambela	1.9 (1.2, 2.9)			2.1 (1.4, 3.1)	**2.7 (1.7, 4.3)**
Harari	1.1 (0.7, 1.7)			1.1 (0.7, 1.7)	0.8 (0.5, 1.4)
Addis Ababa	0.3 (0.2, 0.4)			0.4 (0.3, 0.6)	**0.3 (0.2, 0.5)**
Dire Dawa	0.6 (0.4, 1.0)			0.7 (0.4, 1.0)	**0.5 (0.3, 0.9)**
**Community-level wealth**					
Low	0.5 (0.3, 0.5)			0.6(0.4, 0.8)	**0.7 (0.5, 0.9)**
High	1				
**Community-level education**					
Low	0.4 (0.3, 0.5)			0.6 (0.5, 0.8)	0.8 (0.6, 1.1)
High	1				
**Community-level television exposure**					
Low	0.5 (0.4, 0.6)			0.9 (0.7, 1.2)	1.0 (0.8, 1.4)
High	1				
**Community-level radio exposure**					
Low	0.5 (0.4, 0.6)			0.9 (0.8, 1.3)	1.1(0.8, 1.5)
High	1				

1 = Reference

### Random effects (measures of variation)

Early sexual initiation among female youth varies significantly across each cluster. ICC indicated that 22.6% of the variation in early sexual initiation among female youth was attributed to community-level factors. PCV in the final model shows 42.7% of the variation in early sexual initiation across communities was explained. Likewise, MOR for early sexual initiation among female youth, in the null model was 5.0 which shows the presence of variation across each cluster and it showed a relative reduction in the final model (3.8) (Table 3).

**Table 3:** Measure of variation for early sexual initiation among female youth in Ethiopia, EDHS 2016

Measure of variation	Model-0	Model-I	Model -II	Model-III
Variance	0.9	0.7	0.6	0.5
ICC (%)	22.6	16.9	14.8	14.3
PCV (%)	Reference	30.2	40.6	42.7
MOR	5.0	4.1	3.9	3.8
Model fitness				
Log-likelihood	− 3727.4	− 3152.7	− 3647.3	− 3115.0

*ICC* Intra-class Correlation Coefficient, *PCV* Proportional Change in Variance and *MOR* Median Odds Ratio

## Discussion

The result of the final model showed that individual-level factors: (age, educational status and Chat chewing) and from community-level factors: (region and community-level of wealth) were determinant factors of early sexual initiation in Ethiopia. Cohorts of youth from 19 to 24 years old are more likely to start sex early than cohorts of 15–18 years old. The finding was supported by a study conducted in Wollega, Ethiopia [19]. It was also congruent with studies conducted in Mexico and Korea [4, 7, 36–38]. The possible reason for this association may be due to cultural malpractices like early marriage and abduction which were common but, decreased in the last five years as the data represents at what age they started first sex. Moreover, the difference in the two age-group cohorts may be due to the improvement of youth-friendly health service through time which might increase their knowledge, self-confidence and other sexual issues [39].

As the level of educational attainment increase the risk of early sexual initiation decrease. The finding was supported by studies done in different part of Ethiopia, Alamata [12], Aksum (30), Northern Ethiopia [31], central Tigray (37) and Nekemt [38]. The finding was also supported by research done in South Korea [23]. This might be due to as education increased exposure and access to information regarding the effect of early sexual initiation on their mental and social health. Furthermore, education may bring a behavioral change towards the reduction of risk factors like, substance use which may expose them to early sexual initiation [40]. Moreover, parent-youth communication and supervision might be good for youth who are educated [13, 14, 22, 25, 41].

Chewing Chat was positively associated with early sexual initiation. The finding is consistent with other studies conducted in a different part of Ethiopia [5, 11, 38]. It was also in agreement with studies conducted in Mexico [4], Philippines [17], Korea [42] and Canada [43]. This might be due to the nature of substances to affect individual consciousness and critical thinking about the risk and consequences of early sexual initiation [15, 22, 24, 25, 44]. Moreover, substance users are more subjected to causal sex and they may use it as a means of income for their addiction.

There was a regional variation in age at first sexual initiation. Female youth who lived in Addis Ababa, Dire Dawa, SNNPR, Oromia and Somali region were less likely to initiate sex early as compared to youth who lived in Tigray region. Whereas female youth who were living in Gambella region were more likely to start sex early than living in Tigray region. This might be due to the difference in cultural and religious values and norms across the regions. Social changes, family dynamics and attitude and expression of sexual behavior in youth may be the possible reason for this association [13, 22, 25, 29, 45–47].

When a low proportion of poor people lived in the cluster, the initiation of early sex was decreased. This finding was supported by a study conducted in Kenya [35]. This might be due to rich peoples may have good health-seeking behavior, knowledge on risk factors and parental style. The youth by themselves may have access to different behavioral change communication through different mass media. The above reasons may have an effect on the value and norms of the community towards early sexual initiation and early marriage [9, 16, 45]. Even though the result of this study was more representative than other studies and the model considered different levels of analysis, it is not without limitations. The result may be prone to recall bias because the data were collected from the history of the event.

## Conclusions

After computing multi-level analysis, a cohort of old age, low educational status, ever chewing Chat, region and live in a high proportion of poor community had a statistical association with early sexual initiation among female youth in Ethiopia. Improving universal access to education is important to reduce the prevalence as well as health and health-related complications of early sexual initiation. Advocacy and behavioral change communication among substance user should be an area of concern for different organizations that working on youth reproductive health. Since early sexual initiation differs across community differences, better to develop community sensitive approaches for different communities.

## Data Availability

The datasets used and/or analysed during this study are available from the corresponding author on reasonable request.

## Abbreviations

CSA: Central Statistics Agency
EA: Enumeration Area
ICC: inter cluster coefficient
MOR: Median Odds Ratio
PCV: Proportional Change Variance
